# Sustainable Wheat Protein Biofoams: Dry Upscalable Extrusion at Low Temperature

**DOI:** 10.1021/acs.biomac.2c00953

**Published:** 2022-11-09

**Authors:** Mercedes A. Bettelli, Antonio J. Capezza, Fritjof Nilsson, Eva Johansson, Richard T. Olsson, Mikael S. Hedenqvist

**Affiliations:** † Department of Fibre and Polymer Technology, Polymeric Materials Division, School of Engineering Sciences in Chemistry, Biotechnology, and Health, KTH Royal Institute of Technology, Stockholm10044, Sweden; ‡ FSCN Research Centre, Mid Sweden University, Sundsvall85170, Sweden; § Department of Plant Breeding, SLU Swedish University of Agriculture Sciences, Alnarp, Box 190 Lomma, SE-23422, Sweden

## Abstract

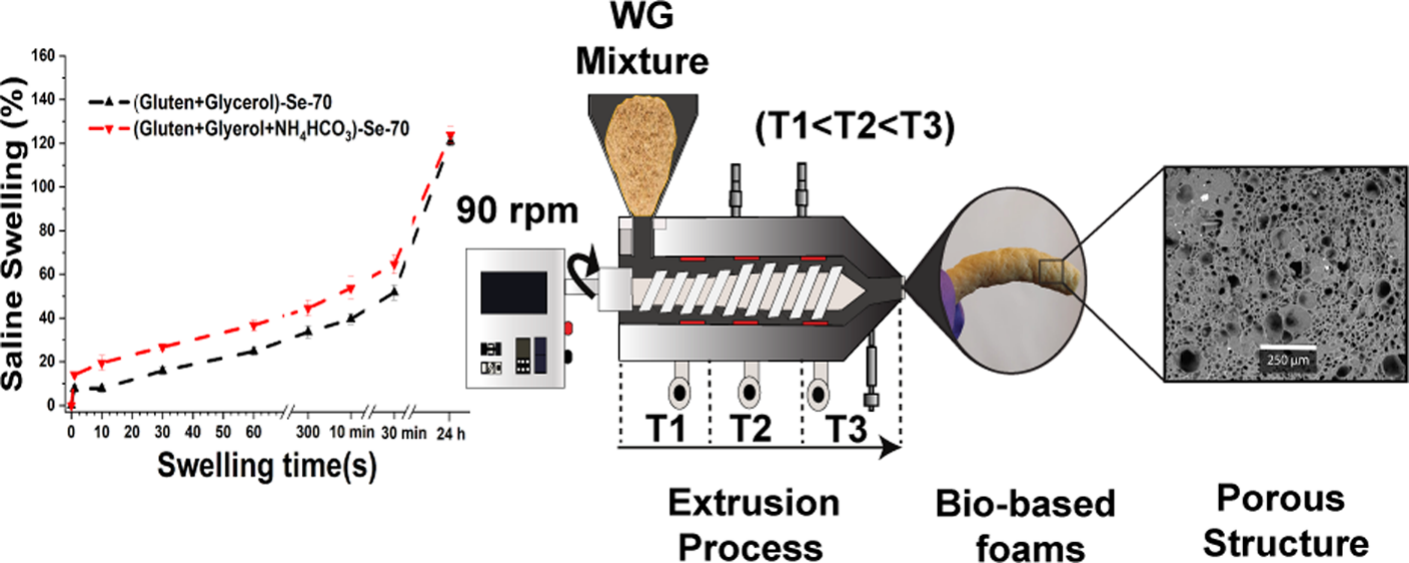

Glycerol-plasticized wheat gluten was explored for producing soft high-density biofoams using dry upscalable extrusion (avoiding purposely added water). The largest pore size was obtained when using the food grade ammonium bicarbonate (ABC) as blowing agent, also resulting in the highest saline liquid uptake. Foams were, however, also obtained without adding a blowing agent, possibly due to a rapid moisture uptake by the dried protein powder when fed to the extruder. ABC’s low decomposition temperature enabled extrusion of the material at a temperature as low as 70 °C, well below the protein aggregation temperature. Sodium bicarbonate (SBC), the most common food-grade blowing agent, did not yield the same high foam qualities. SBC’s alkalinity, and the need to use a higher processing temperature (120 °C), resulted in high protein cross-linking and aggregation. The results show the potential of an energy-efficient and industrially upscalable low-temperature foam extrusion process for competitive production of sustainable biofoams using inexpensive and readily available protein obtained from industrial biomass (wheat gluten).

## 1 Introduction

Polymer foams are important in industrial applications due to their unique properties, including high affordability, good chemical inertness, low density, high strength, high flexibility, and high thermal resistivity. This enables the production of materials for cushioning, damping, impact resistance, and thermal-electrical- and sound insulation.^1,2^ Commodity polymer foams, based on, for example, polystyrene, polyether, polyester, polyurethane, polypropylene and polyethylene, are most frequently used in engineering applications.^3,4^ However, these materials are produced from fossil-based resources (oil) and are unsuitable for composting because they do not degrade naturally in the environment. Hence, there is a strong incentive to find sustainable alternatives to the traditional synthetic polymers. One alternative is to use wheat gluten (WG), which is readily foamable, refer to its bread leavening properties.^5,6^ It is also a coproduct of starch-ethanol production and, in some parts of the world, considered a byproduct.^1^ Therefore, especially where there is a surplus of it, alternative nonfood applications are considered for WG.^6^ Because it is biodegradable and a source of nutrition to soil and living species, it is not likely to yield the same microplastic problems after use, as in the case of today’s commercial polymers.

The strong cohesive properties and viscoelastic nature of WG^7,8^ make it a potential material option for producing foams through conventional extrusion.^9−12^ The production of WG foams from aqueous solutions through lyophilization has been reported^9,13,14^ and also using other techniques like microwave heating.^10,15,16^ Moreover, WG foams with a range of built-in features have been made, including improved electric and thermal conductance,^13,17,18^ superabsorbency,^9,19−21^ and microbial resistance.^14,22^

Extrusion is a promising technology for transforming proteins rapidly into consumer products. During extrusion, the protein is subjected to several restructuring mechanisms involving denaturation of the protein and the formation of new covalent and noncovalent bonds.^23^ The rheological properties of the materials during processing, and the mechanical properties of the produced product can be tailored using different contents of plasticizer, for example, glycerol.^9,24−26^ To achieve porosity during processing, chemical blowing agents (CBA) are often used to produce extruded foams. A popular CBA is sodium bicarbonate (SBC), a leavening agent used in the bakery industry, which has recently been used to extrude expanded/porous wheat gluten materials.^27^ The heat-induced decomposition of the agent generates carbon dioxide, which nucleates and produces bubbles at the exit of the die.^2,25^ Ammonium bicarbonate (ABC) is another common leavening agent. It reacts during the batter mixing and decomposes fully at a lower temperature (∼60 °C) than SBC, the latter gradually decomposing from 106 °C.^28,29^ ABC releases carbon dioxide, ammonia and water, without requiring an acid for its decomposition. It is used in smaller baked goods to allow rapid evaporation of the generated ammonia.^29^ Its low decomposition temperature opens up for the foaming of protein materials like wheat gluten below the temperature region where the protein aggregates, the latter leading to a large increase in melt viscosity.

In this article, the main objective was to evaluate the possibility to produce sustainable biofoams by dry low-temperature extrusion foaming of wheat gluten. Thererfore, the effects of using ammonium bicarbonate in the foam extrusion process was elucidated and compared with the performance of sodium bicarbonate, both environmentally friendly blowing agents. The hypothesis was that ABC would lead to a more effective formation of a foamed extrudate than SBC and that the protein could be extruded at a lower temperature, thereby reducing the risk of heat-induced protein aggregation and reducing the energy needed for direct heating in the extrusion process. The extrusion was performed in the presence of a plasticizer (glycerol) to obtain soft biofoams.

## 2 Experimental Section

### 2.1 Materials

Wheat gluten powder was supplied by Lantmämmen Reppe AB, Sweden, as a coproduct from the industrial wheat starch production/extraction. The powder consisted of 85.2 wt % wheat gluten protein (*N* × 6.25), 5.8 wt % wheat starch, 1.2 wt % lipids, 0.9 wt % ash, and at ambient conditions, about 7 wt % water. Glycerol (ACS reagent ≥99.5%), sodium bicarbonate (SBC, NaHCO_3_, ACS ≥98%) and ammonium bicarbonate (ABC, NH_4_HCO_3_, ACS ≥98%) were provided by Sigma-Aldrich, Sweden. The polylactic acid (PLA, Ingeo 4042D, density: 1240 kg/m^3^, melting point: 150 °C) material was obtained from Nature Works (U.S.A.), and the low-density polyethylene (LDPE, FA6224, density: 922 kg/m^3^, melting point: 111 °C) was obtained from Borealis (Sweden). The melt flow index of the PLA is 6 g/10 min (210 °C, 2.16 kg) and that of the LDPE is 6 g/10 min (190 °C, 2.16 kg). The LDPE and PLA materials were selected in order to compare the energy required to process WG with those of two commonly extruded commercial materials.

### 2.2 Foam Preparation

**Figure 1:**
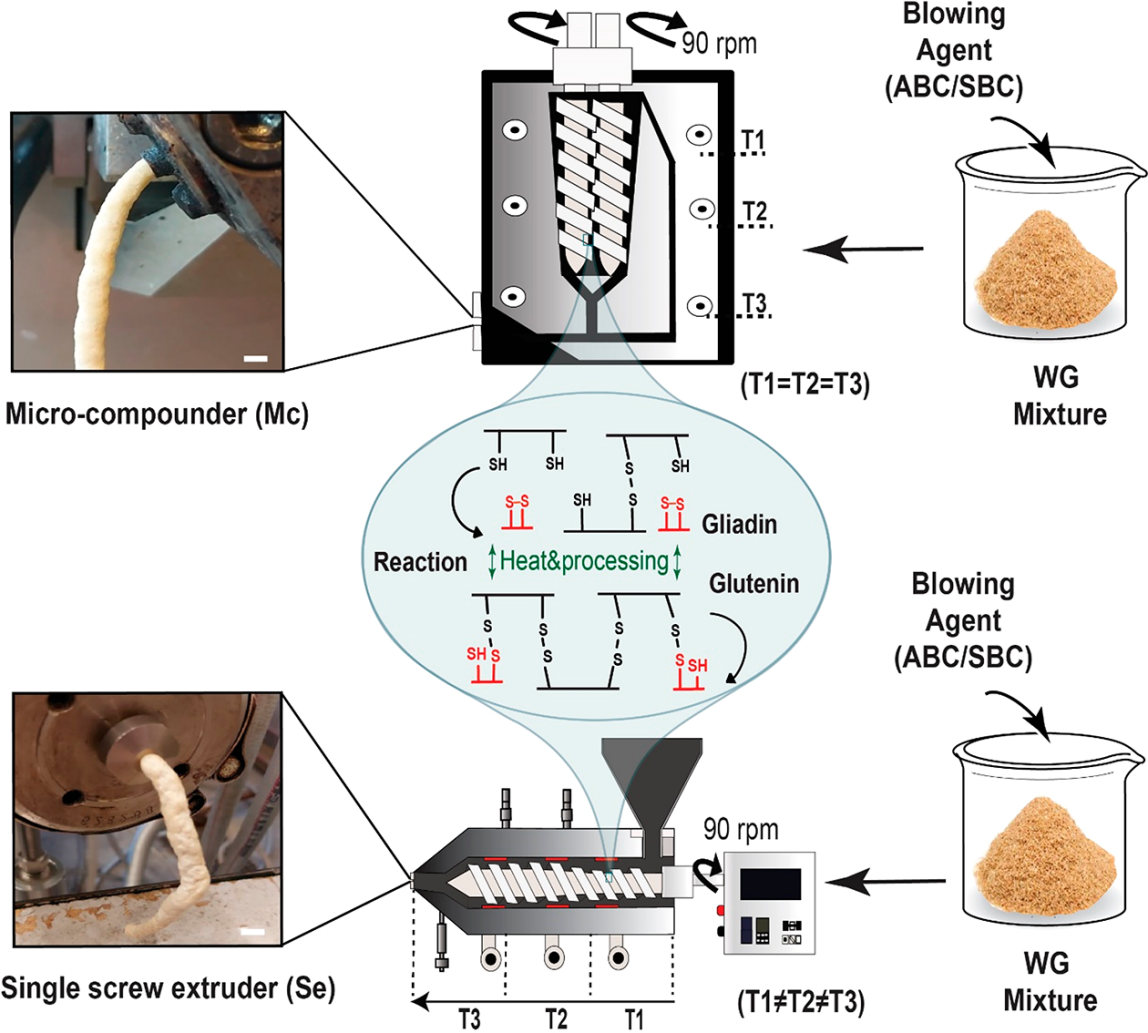
Illustration of the extrusion with microcompounder (Mc) and single-screw extruder (Se). The extrudate in the images is the ABC containing material. The scale bars in the images are 2.8 mm (Mc) and 3.8 mm (Se) long, respectively.

For a 50 g batch of WG/glycerol, 35 g (70 wt %) of WG powder was manually mixed with 15 g (30 wt %) of glycerol until a homogeneous mixture was obtained. The 70/30 gluten/glycerol weight ratio was selected based on previous work, which showed that this composition yielded a combination of mechanically flexible and ductile films and good extrudability.^30^ When using the foaming agents SBC and ABC, 2.5 g (5 wt %) of these were added during the mixing, and the WG content was lowered to 32.5 g, following the previous optimization work with SBC.^9^ The mixture was processed with two different types of equipment: I, a conical fully intermeshing and corotating double-screw microcompounder (DSM Xplore 5 cc, The Netherlands); II, a single screw extruder (Brabender Do-Corder C3, Germany). The microcompounder contained screws with an L/D ratio of 8, and a compression ratio of 3.3. The single screw extruder contained a screw with an L/D ratio of 20 and a compression ratio of 2.5. In the microcompounder, all heating zones were set to the same temperature: 70 °C when using ammonium bicarbonate (ABC) and 120 °C when using sodium bicarbonate (SBC). The screw speed was 90 rpm, and a circular die with a diameter of 2.8 mm was used. For the materials manufactured by the single screw extruder, a screw speed of 90 rpm was used and the heating zones, from the feed zone to the die, were set at 50–60–70 °C (ABC) and 100–110–120 °C (SBC). The specific temperatures were selected to gradually initiate the ABC and SBC reactions toward the die region. It was also observed that using a too high temperature close to the hopper would make the material sometimes go backward into the hopper. A circular die with a 3.8 mm diameter was used in the single-screw extruder. The extrudates were dried in a forced-air oven at 40 °C for 24 h after the extrusion, and then stored inside a desiccator with silica gel for at least 1 week before the tests. The full description of the samples is given in Table 1; as an example, the sample prepared with WG, glycerol and ammonium bicarbonate were named WG/G/5ABC-Mc and WG/G/5ABC-Se, when fabricated in the microcompounder (Mc) and single-screw extruder (Se), respectively. The sample production is illustrated in Figure 1.

**Table 1:** Sample Nomenclature

sample name^a^	WG (wt %)	G (wt %)	ABC (wt %)	SBC (wt %)	processing conditions (° C)^b^
WG/G-Mc-70	70	30			70
WG/G/5ABC-Mc-70	65	30	5		70
WG/G-Se-70	70	30			70
WG/G/5ABC-Se-70	65	30	5		70
WG/G-Mc-120	70	30			120
WG/G/5SBC-Mc-120	65	30		5	120
WG/G- Se-120	70	30			120
WG/G/5SBC-Se-120	65	30		5	120

a Mc: Microcompounder; Se: Single-screw extruder.

b Se processing conditions: 50–60–70 °C (±2 °C); SBC processing conditions: 100–110–120 °C (±5 °C). All the samples were extruded at a rotational screw rate of 90 rpm.

### 2.3 Specific Mechanical Energy (SME)

The specific mechanical energy was estimated using eqs 1 (microcompounder) and 2 (single-screw extruder).^2^
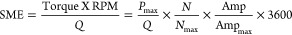
1
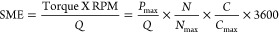
2*P*_max_ and *Q* are the maximum power of the extruder’s motor (kW) and the material input (kg/h). *N* is the screw rotation rate (rpm), and *C* is the measured torque of the motor (Nm). The motor’s maximum power was 0.4 and 3.3 kW, and the maximum screw rotation rate was 400 and 250 rpm for the mini-compounder and single-screw extruder, respectively. In the case of the microcompounder, torque values were not provided. However, the torque can be indirectly estimated from the amount of energy or the applied electric current required to run the screw.^31^ Therefore, the power during the process was used. Amp is the applied electrical current (Amp), and Amp_max_ is the maximum possible current in the equipment. The number 3600 yields the SME in eqs 1 and 2 in kJ/kg

### 2.4 Density

The sample density and porosity were determined in a sequence of measurements and calculations. The volume of the solid matrix in the foam sample (*V*_m_) was calculated from the sample mass in air (*m*_a_) (using a Mettler Toledo AL104 balance (Switzerland)) divided by the solid gluten/glycerol density ρ_m_ = 1290 kg/m^3^. The latter represents the value of solid WG (ρ_WG_ = 1300) with 30% glycerol (ρ_G_ = 1260).^32^ By the use of an Archimedes accessory to the balance, the volume of closed pores (*V*_CP_) could be calculated from the sample mass in air (*m*_a_) and in a liquid (*m*_L_), also knowing the density of the liquid (ρ_L_) and the volume of the matrix (*V*_m_; eq 3). In most cases limonene was used as the liquid (ρ_L_ = 842 kg/m^3^), but for samples with a density lower than limonene, *n*-heptane (ρ_L_ = 684 kg/m^3^) was used. These organic liquids were poor solvents and did not solubilize the material. The volume of open pores (*V*_OP_) was calculated using eq 4 by first determining the mass of the wet sample (*m*_w_). It was measured after only 1 s immersion in the liquid to obtain the capillary uptake, considering that the pores were rapidly filled with the liquid and no uptake of the hydrophobic liquid occurred in the polar material during this time period.
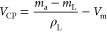
3
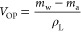
4From the ratio of these volumes to the total volume (*V*_total_ = *V*_OP_ + *V*_CP_ + *V*_m_), the porosity of open and closed pores was calculated. The density was finally calculated as *m*_a_/*V*_total_.

### 2.5 Scanning Electron Microscopy (SEM) and Energy-Dispersive X-ray Spectroscopy (EDS)

The morphology of the samples was analyzed using a Hitachi TM-1000 Tabletop SEM 10 kV voltage, (Japan). The sample cross-section surface was obtained by breaking the specimen after immersing it in liquid nitrogen for 5 min. The cryo-fractured pieces were placed onto aluminum specimen holders using conductive carbon tape. The extrudate sample was frozen at −25 °C, cryo-fractured, and analyzed to evaluate the sample’s structure after saline swelling for 24 h using a Hitachi S-4800 field emission scanning electron microscope FE-SEM, (Japan). A voltage of 3 kV and a current of 10 μA were used, and the WG foams were sputtered with a palladium/platinum with a conductive layer of 1–2 nm using an Agar High-Resolution Sputter Coater (model 208HR). EDS (Oxford Instrument) attached to the FE-SEM was used to determine the presence of elements using a voltage of 5.5 kV and a current of 20 μA. The pore size cross sections of the samples were measured using the image analysis program ImageJ, based on at least 50 measurements.

### 2.6 Liquid Swelling Measurement

The swelling/uptake capacity (SC) of the WG extrudate was measured by placing the sample into an empty teabag and then immersing it in the liquid. The sample, with a weight of about 200 mg, was cut out along the extrudate cross-section so as to represent the whole extrudate geometry. The tea bag was used to ensure that no material was lost in the process. A sample was placed into a plastic tea bag (*W*_d_), then hooked to a glass rod and immersed in beakers containing saline solution (0.9 wt % NaCl in water), or limonene, for different times (up to 24 h for saline and up to 30 min for limonene). The bags with the material were hung for 10 s and then touched gently against tissue paper for 10 s to remove excess liquid after the SC test. The wet sample was removed intermittently from the tea bag and weighed (*W*_i_). The SC results were reported as grams of absorbed liquid per gram of dry material on a percentage scale and presented as the average of triplicates according to eq 5. The empty bags and extrudates were kept in a desiccator with silica gel for a minimum of 48 h before the SC test.
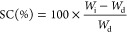
5

To determine the saline uptake kinetics/diffusivity and to have a strict radial diffusion, samples with a length 10× the diameter were used.^33^ Triplicate specimens were used for each sample type.

### 2.7 Fourier-Transform Infrared Spectroscopy (FTIR)

FTIR spectra were obtained by using a PerkinElmer Spectrum 100 (U.S.A.), equipped with a triglycine sulfate (TGS) detector and a Golden Gate unit (ATR, Graseby Specac LTD, England). The scanning resolution was 4.0 cm^–1^, and 16 scans were used. The spectra in the region from 1700 to 1580 cm^–1^ were deconvoluted using an enhancement factor γ of 2 and a smoothing filter of 70% and then baseline-corrected.^10^ The peak deconvolution was obtained with a PerkinElmer spectrum, and the peak resolution was obtained with the Origin software. The resolved peaks were all normalized to the total Amide I band absorbance. All the samples were kept in a desiccator with silica gel for at least 1 week before the FTIR measurements.

### 2.8 Size-Exclusion High-Performance Liquid Chromatography (SE-HPLC)

The protein solubility was assessed by SE-HPLC Waters 2690 separations module and a Waters 996 photodiode array detector (Waters, U.S.A.) using a Biosep-SEC-S4000 (300 Å ∼ 4.5 mm) with a prefilter SecurityGuard GFC 4000 (Phenomenex, U.S.A.) using a three-step extraction procedure, all at room termperature, described fully in Gällstedt et al.^11^ It will be described only briefly here. In the first step, 16 mg of each foam sample was suspended in 1.4 mL of 0.5 wt % SDS buffer (pH 6), and the supernatant (SN) was obtained after a centrifugation at 2000 rpm, followed by 5 min agitation. The second extraction (Ext. 2) was performed by resuspending the residual sample after the first extraction in a new SDS buffer solution and sonicating it in a ultrasonic disintegrator for 30 s. The third extract (Ext. 3) was obtained by resuspending the residual sample after the second extraction in a new SDS buffer solution and sonicating it over a longer period of time (30 + 60 + 60 s). Three replicates per formulation were used. The SE-HPLC analysis was performed, using a 0.2 mL/min of an isocractic flow consisting of 50% acetonitirile, 50% Millipore water, and 0.1% trifluoracetic acid.

## 3 Results and Discussion

### 3.1 Foam Structure

**Figure 2:**
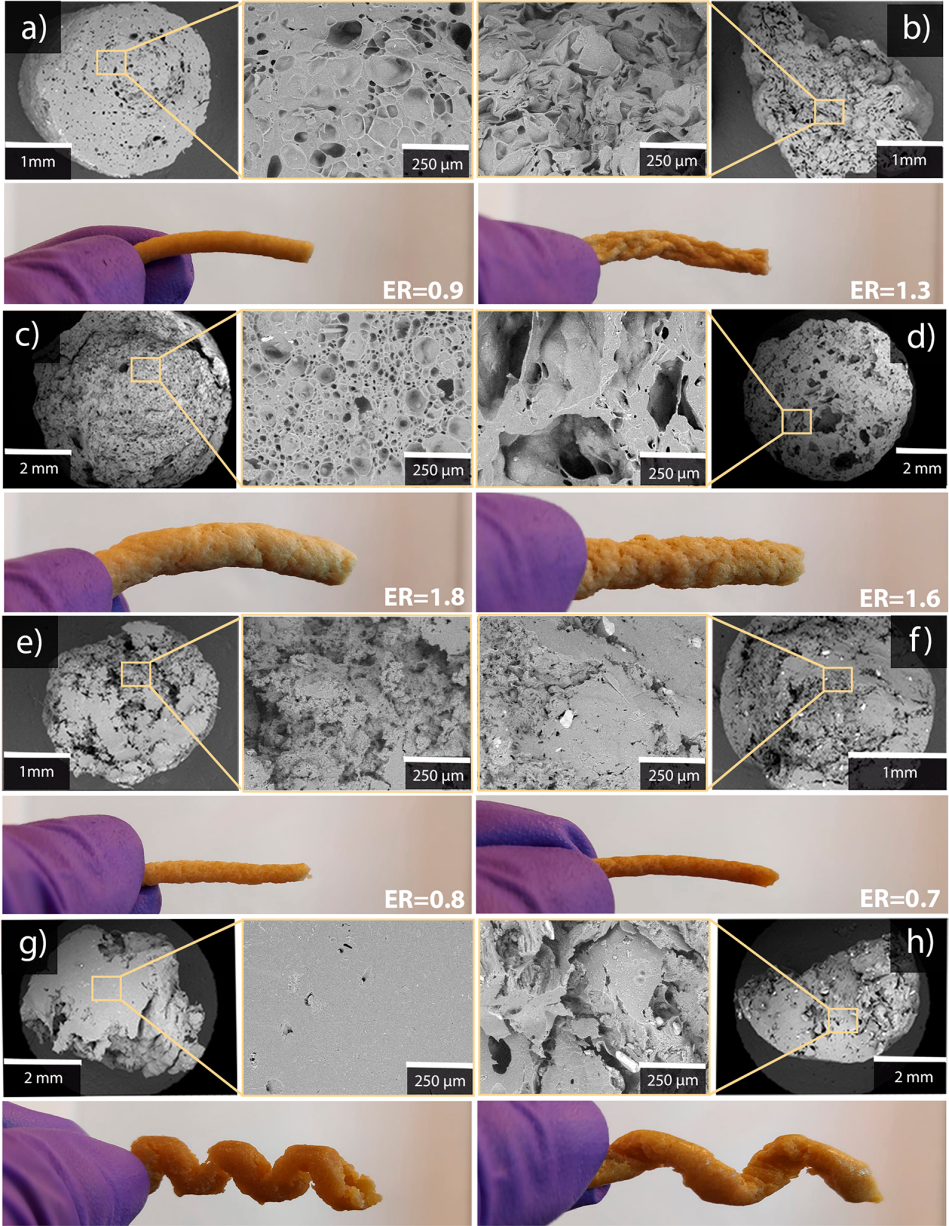
SEM cross sections and the appearance of extruded samples after drying: (a) WG/G-Mc-70, (b) WG/G/5ABC-Mc-70, (c) WG/G-Se-70, (d) WG/G/5ABC-Se-70, (e) WG/G-Mc-120, (f) WG/G/5SBC-Mc-120, (g) WG/G-Se-120, and (h) WG/G/5SBC-Se-120. ER refers to the expansion ratio.

The different extrudates, after drying in the desiccator, are shown in Figure 2. Overall, all samples showed a porous structure, although the porosity and pore structure varied. In some cases, a core–shell structure could be observed with a denser outer part. The sample extruded without a foaming agent in the microcompounder at 70 °C (WG/G-Mc-70) showed a porous cross-section (Figure 2a). This was most likely due to the presence of moisture. Even though the dry WG powder was mixed with the constituents just before feeding it to the extruder, the powder particles were small enough to rapidly take up moisture (average size of ca. 30 μm, Figure S1). The sample did, however, not experience any visible expansion after the die. The expansion ratio (ER, the ratio of the sample diameter and die diameter) of the dried material was also below 1. Hence, the material experienced a slight collapse in the radial direction as the low viscous melt left the die. The density was relatively high (910 kg/m^3^), and the total porosity was consequently relatively low (30%), with an average pore size of 70 μm (Table 2). With the use of ammonium bicarbonate (WG/G/5ABC-Mc-70), the density decreased (770 kg/m^3^), both the closed and the open porosity increased, and the average pore size was somewhat larger than in the WG/G-Mc-70 sample. However, the SEM image indicated a collapsed pore structure, which could also be observed visually by the uneven surface (Figure 2b). Hence, without the collapse, an even lower density would be obtained. We noted that this collapse occurred not only during the drying of the sample in the desiccator but also when the sample was instead conditioned at 50% relative humidity after extrusion. Nevertheless, the final expansion ratio was still higher with ABC than without it (compare Figure 2a and b).

**Table 2:** Physical Properties of the Different Extrudates

sample name		density (kg/m^3^)	open porosity (%)	closed porosity (%)	total porosity (%)	pore size^a^ (μm)
	WG/G solid material	1290				
(a)	WG/G-Mc-70	910 ± 19	11	19	30	70 ± 30
(b)	WG/G/5ABC-Mc-70	770 ± 25	17	23	40	80 ± 55
(e)	WG/G-Se-70	700 ± 22	4	41	45	70 ± 60
(f)	WG/G/5ABC-Se-70	810 ± 10	8	29	37	180 ± 30
(c)	WG/G-Mc-120	910 ± 15	27	2	29	90 ± 70
(d)	WG/G/5SBC-Mc-120	1080 ± 42	15	2	17	70 ± 30
(g)	WG/G-Se-120	1100 ± 26	15		15	NM
(h)	WG/G/5SBC-Se-120	1030 ± 35	11	9	20	NM

a The pore size distribution was obtained from measurements on SEM images; values and standard deviations (±values) were based on a minimum of 50 measurements on each sample. NM: Not measured, difficult to resolve the pores. The letters in the beginning of the rows refer to the letters in Figure 2.

The extrudate produced without ABC in the single-screw extruder (WG/G-Se-70) experienced a larger expansion ratio and a significantly higher closed cell porosity than any of the two materials produced in the microcompounder (Figure 2c, Table 2). Interestingly, in the presence of ABC, the single-screw extrudate (WG/G/5ABC-Se-70) did not yield a lower density than that produced without ABC. This was due to a greater collapse of the ABC expanded sample (note the more uneven surface of this extrudate (Figure 2d) compared to that in Figure 2c). Note, however, the still very large pores in the ABC sample, despite some collapse. The densities of the samples presented so far fall within the group of high-density foams,^34,35^ and are higher than in common low-density foams of polyurethane, polyethylene, and polystyrene.^17,36−38^

The samples extruded at the higher temperature (maximum 120 °C), where sodium bicarbonate was added as a possible foaming agent, behaved quite differently from those produced at the lower temperature (maximum 70 °C). Many proteins, including gluten, contain cysteine, which contributes to a thiol group that can form intra- and intermolecular disulfide bonds. Even though the cysteine content in gluten is relatively low (only a few percent of the amino acids present), the disulfides still determine to a large extent the dough/melt properties.^39^ Whereas the viscosity decreases with temperature for pure thermoplastics in the molten state (shear thinning), the formation of new disulfide bonds or rearrangement of existing disulfide bonds from intra- to intermolecular bonds in gluten leads to an increase in viscosity (protein polymerization/cross-linking) at high temperatures, leading to protein aggregation.^40^ This is one reason for the different structures and densities of the gluten/glycerol samples extruded in the microcompounder at 70 and 120 °C (compare, for example, Figure 2a,e). The foam extruded at 120 °C in the microcompounder (WG/G-Mc-120) obtained by far the highest open porosity of all samples (Figure 2e). In this sample, as in all samples produced at the higher temperature, the foam structure was quite nonuniform with less easily observable cells. This was also the case with the presence of the foaming agent SBC. Whereas it is possible to readily foam gluten/glycerol at 120 °C with sodium bicarbonate in an open environment (oven), with or without water, the situation is very different in extrusion here (under large hydrostatic pressure; Figure 2f). Residual crystals of sodium bicarbonate or the reaction products, sodium carbonate and carboxylates, were also observed (bright particles in Figure 2f). When extruded in the single-screw extruder, the extrudates, with or without SBC, came out in a spiral shape (Figure 2g,h). This indicated that the samples were mainly transported through the extruder with a low degree of mixing/compounding, a consequence of high protein aggregation. The sample without SBC (WG/G-Se-120) showed an almost pore-free structure (Figure 2g), and it had the lowest porosity of all samples (Table 2). The pore structure of the SBC sample was, as in the case of the microcompounded sample (Figure 2d), not showing a typical foam cell structure but the presence of bright particles (Figure 2h).

Ammonium bicarbonate decomposes into carbon dioxide, water, and ammonia. Sodium bicarbonate reacts with itself into carbon dioxide, water, and sodium carbonate, the latter being strongly alkaline. The alkalinity has been shown to oxidize thiol groups into more disulfide bonds.^41^ This would yield a more aggregated protein that seemed to predominate over the CO_2_-induced generation of pores in extrusion. This resulted in the overall lower porosity and higher density in the presence of SBC, rather than with ABC. The overall higher aggregation at 120 °C also yielded a lower degree of compounding/mixing and, therefore, the spiral-shaped samples in the single-screw extrusion. In the previous work, to reduce the protein aggregation, SBC containing materials were also extruded at lower temperature (80 °C at the die section) using a large content of water.^9^ However, also in this case, the foaming during the extrusion was low, yielding a material with high density and very low porosity. The lower efficiency of foaming at the lower temperature in combination with the hydrostatic pressure in the extruder were probably the reasons for this.

### 3.2 Protein Structure

**Figure 3:**
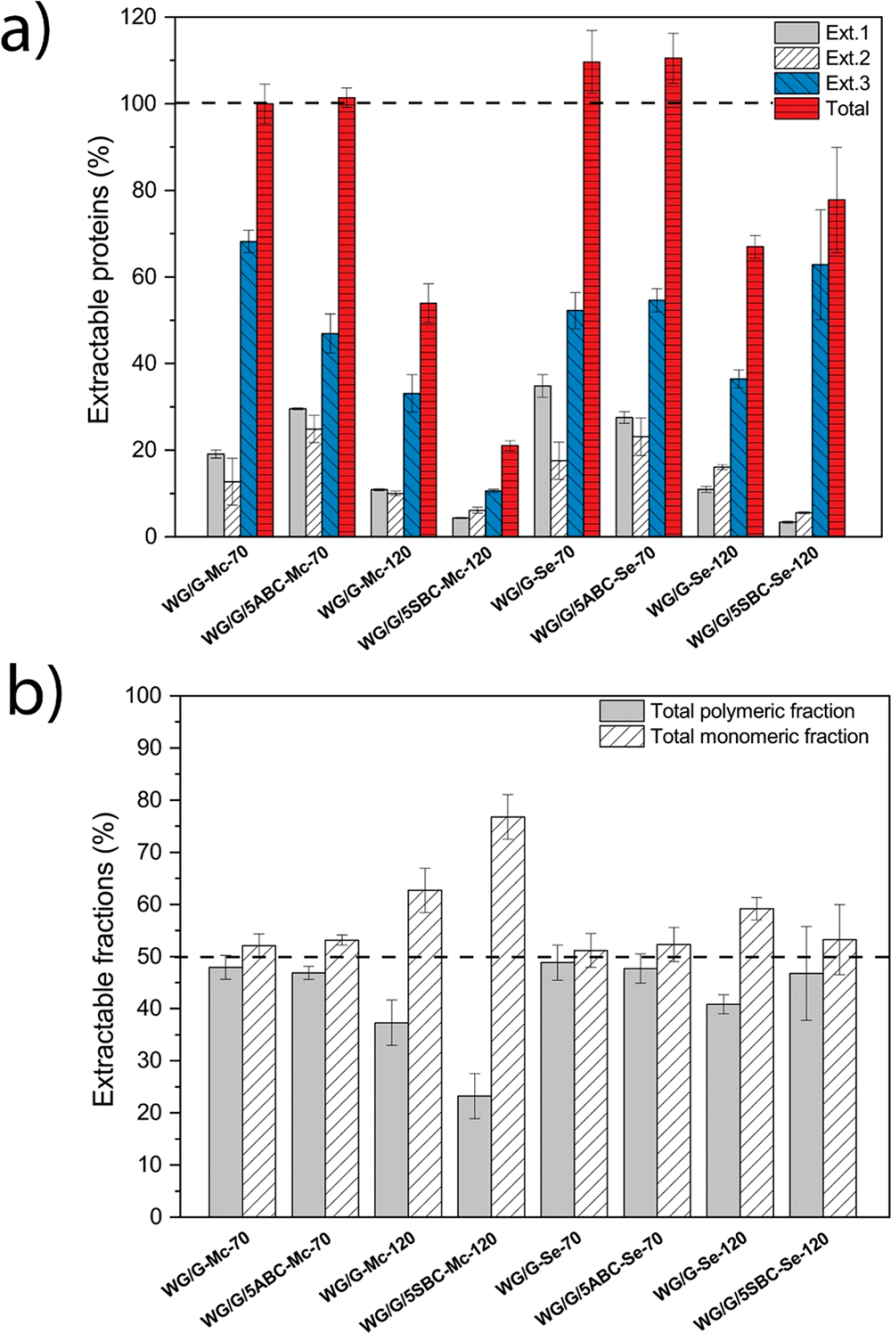
SE-HPLC results: (a) Relative amount of extractable proteins in the three extraction steps and (b) relative amount of total polymeric (PP) and monomeric (MP) proteins extracted. The total extractable protein was normalized to that of the WG foam processed at 70 °C in the microcompounder.

SE-HPLC is a useful method for investigating protein solubility behavior and associated protein aggregation.^1,13,21,42^ It was also used to estimate the relative degree of aggregation in the different samples. The protein solubility, when using only the surfactant (SDS) to cleave the secondary bonds, was lowest for the samples extruded at the higher temperature, showing a more extensive protein (disulfide) cross-linking/polymerization and aggregation (Figure 3a). This was particularly the case in the presence of SBC, as explained by its alkaline reaction product. The following extractions with a combination of SDS and sonication, increased further the protein solubility. Still, the total protein solubility was always lower at the higher temperature, and lowest for the SBC sample extruded with the microcompounder. These results are in-line with the microstructures shown in Figure 2. The total solubility at the lower extrusion temperature was the same with and without ABC, showing that ABC did not contribute to protein aggregation. The extracted amount of monomeric proteins (proteins/polypeptides of lower molar mass that are eluted after 14.3 min in the SE-HPLC experiment) was always higher than the extracted amount of polymeric proteins (proteins eluted after 14.3 min; Figure 3b). The difference was, however, especially large at the higher extrusion temperature and largest for the SBC sample extruded in the microcompounder. Hence, the low solubility of that system was due mainly to the low content of extracted polymeric protein, a consequence of the high degree of polymerization/cross-linking occurring in the extrusion.

### 3.3 Foam Molecular Structure

**Figure 4:**
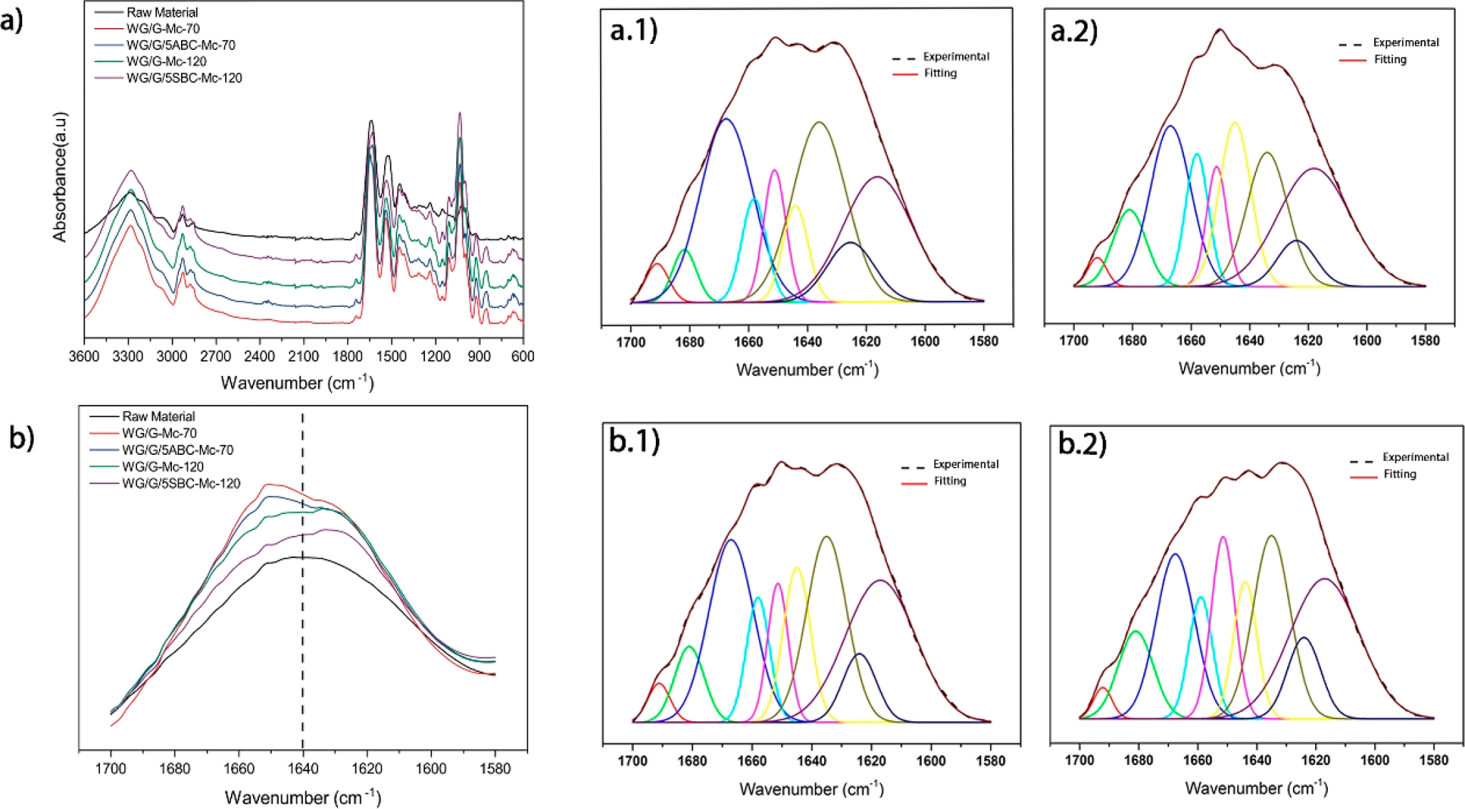
(a) Full IR spectra and (b) the amide I region of samples produced with the microcompounder. The deconvoluted amide I region with the corresponding fitted curves for (a.1) WG/G-Mc-70, (a.2) WG/G/5ABC-Mc-70, (b.1) WG/G-Mc-120, and (b.2) WG/G/5SBC-Mc-120.

FTIR spectra of the samples manufactured with the microcompounder is shown in Figure 4. All samples showed a change in the amide I region (1700–1580 cm^–1^) profile compared to that of the pure protein material, indicating a change in the secondary molecular structure (Figure 4b). A clear difference in the total intensity profile in this region was also observed due to the temperature effect; the samples extruded at the higher temperature had spectra peaking in the 1640–1620 cm^–1^ region, whereas those extruded at the lower temperature had spectra peaking at higher wavenumbers. This type of difference is due to the relative content of different protein conformations due to different degrees of protein aggregation. With the use of the deconvolution and resolution procedure of the different IR peaks/bands contributing to the total spectra (Figure 4 and Table 3),^43,44^ the content of strongly bonded β-sheets was determined to be higher after the Mc extrusion at the higher temperature, indicating a higher degree of aggregation, in-line with the SE-HPLC data above. The same observations were also made for the samples produced with the single screw extruder (Figure S2 and Table S1). Besides the trend in the strongly bonded β-sheets, it is also observed that the content of β-turns was always higher without ABC and SBC (Tables 3 and S1).

**Table 3:** Content of Different Molecular Features from IR Spectra in the Amide I Region

method I: microcompounder					
λ (cm^–1^)	assignment	WG/G-Mc-70 (%)	WG/G/5ABC-Mc-70 (%)	WG/G-Mc-120 (%)	WG/G/5SBC-Mc-120 (%)
1618, 1625	β-sheets strongly bonded	28.1	28.6	29.9	31.4
1634, 1681	β-sheets weakly bonded	26.7	22.2	23.7	24.9
1644, 1651, 1658	α-helixes and random coil	19.4	29.6	24.3	26.3
1667, 1692	β-turns	25.7	19.6	22.1	17.5

### 3.4 Specific Mechanical Energy (SME)

The estimated specific mechanical energy associated with the two extrusion/processing methods is presented in Table 4. A direct comparison of the SME for the two processing methods should be made with caution, since they have been estimated in different ways (eqs 1 and 2). Nevertheless, the fact that the average temperature was lower in the single-screw extruder (using a temperature profile) increased the mechanical energy needed. Note the decrease in SME with increased temperature (compare 70 and 120 °C data). In addition, the effective shear rate was higher in the microcompounder that, because of the strong shear thinning behavior of WG/G, reduced the viscosity compared to in the single-screw extruder and consequently reduced the SME.^45−47^ To assess the determined size of the SME of the WG materials in relation to commonly extruded commercial materials, an extrusion grade LDPE was compounded in the microcompounder at the same high-temperature conditions (120 °C) as the gluten material. It required a higher SME than any of the two gluten materials; in fact, its SME was close to that of the gluten material processed at a lower temperature (70 °C). As a further comparison, we compounded an extrusion grade PLA (PLA is a commonly extruded biobased and biodegradable commercial polymer) in the microcompounder. It turned out, however, that it had the highest SME of all samples due to its required high extrusion temperature. To conclude, considering both the direct heating and the SME, the processing of the gluten material needed less energy than that of both the LDPE and PLA, which is promising when considering gluten to a be a future replacement of these.

**Table 4:** Specific Mechanical Energy (SME)

samples	*T*^a^ (°C)	Mc, SME^b^(kJ/kg)	Se, SME^b^(kJ/kg)
WG/G	70	343	1043
WG/G/5ABC	70	253	1217
WG/G	120	142	630
WG/G/5SBC	120	213	563
LDPE	120	319	
PLA	180	426	

a Die temperature and/or max temp in the process.

b Specific mechanical energy for Mc: microcompounder and Se: single screw extruder. The output rates were 0.14 and 0.26 kg/h for the ABC and SBC systems, respectively.

### 3.5 Liquid Swelling/Uptake Characteristics

Liquid swelling/uptake characteristics can reveal the structural features of a porous material. As mentioned above, limonene was used here to assess the open pore interconnectivity and capillary action. The uptake of the hydrophobic limonene is dominated by capillary action since the liquid fills the pores without any significant/measurable absorption into the hydrophilic WG material.^24,35^

**Figure 5:**
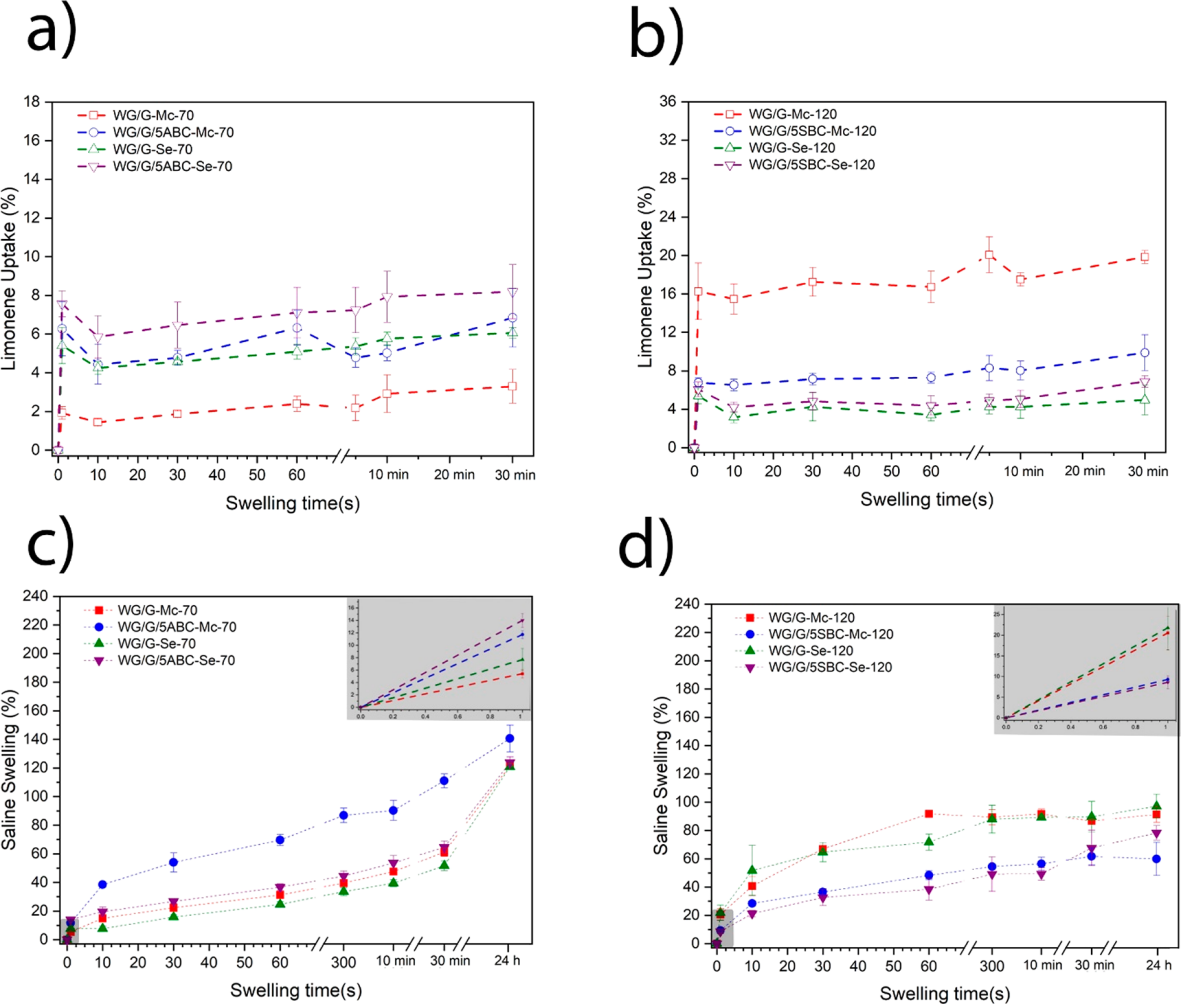
Liquid uptake in the samples; limonene (a) and saline (b) in the ABC system and limonene (c) and saline (d) in the SBC system. The gray regions represent the rapid uptake (0–1 s).

All samples showed a rapid uptake (within 1 s) and no sizable further uptake within 30 min (Figure 5a,b). This behavior is typical of capillary action. In the low extrusion temperature (ABC) system, the highest uptake was observed for WG/G/5ABC-Se-70 (ca. 8 wt %) and the lowest for WG/G-Mc-70 (ca. 2–3 wt %). The latter result may be explained by it having the highest density of the four materials and in the former case it may be associated with it having the largest average pore size (180 μm, Table 2). In the high extrusion temperature system, WG/G-Mc-120 showed by far the largest limonene uptake (ca. 16 wt % after 1 s), whereas the lowest uptake was observed for WG/G-Se-120. However, the difference was not significantly different from the uptake in WG/G/5SBC-Se-120 (Figure 5b). The high uptake by WG/G-Mc-120 was in accordance with its high open porosity (Figure 2e and Table 2). Also, the low uptake in WG/G/Se-120 was in accordance with the low overall porosity (Figure 2g and Table 2).

To investigate the behavior of the foam when immersed in a liquid that is, apart from filling the pores, also absorbed in the foam walls, the saline solution was chosen. The ions in saline lead, by osmotic effects, to a significantly lower uptake than for pure water, but it is an important standard measurement liquid for potential absorbent materials.^35^ Overall, the saline uptake after 24 h was lowest in the high-temperature extrusion system, most likely due to the proteins more extensive polymerization/cross-linking than in the low-temperature system (Figure 5c,d). In fact, the lowest uptake was observed in the presence of SBC, is in accordance with the low protein solubility after the SDS treatment (SE-HPLC, Figure 3a). In contrast to limonene, the saline was also absorbed into the protein cell walls and entered the closed-cell space.^48^ Hence, the rapid uptake of saline, i.e., within 1 s, was a combination of capillary effects and cell wall sorption. Nevertheless, in the low-temperature extrusion system, the limonene and saline uptake showed similar results in that the highest rapid uptake was observed for WG/G/5ABC-Se-70 and the lowest rapid (1 s) uptake was observed for WG/G/Mc-70 (inset in Figure 5c). The highest saline uptake at times longer than 1 s was observed for WG/G/5ABC-Mc-70, probably due to the gradual expansion of the collapsed pore structure (Figure 2b). In the high extrusion temperature system, the rapid (1 s) uptake was highest for the samples without SBC, as also observed at longer uptake times (Figure 5d, inset). As mentioned above, this is probably due to the more extensive polymerization/cross-linking in the presence of SBC.

**Figure 6:**
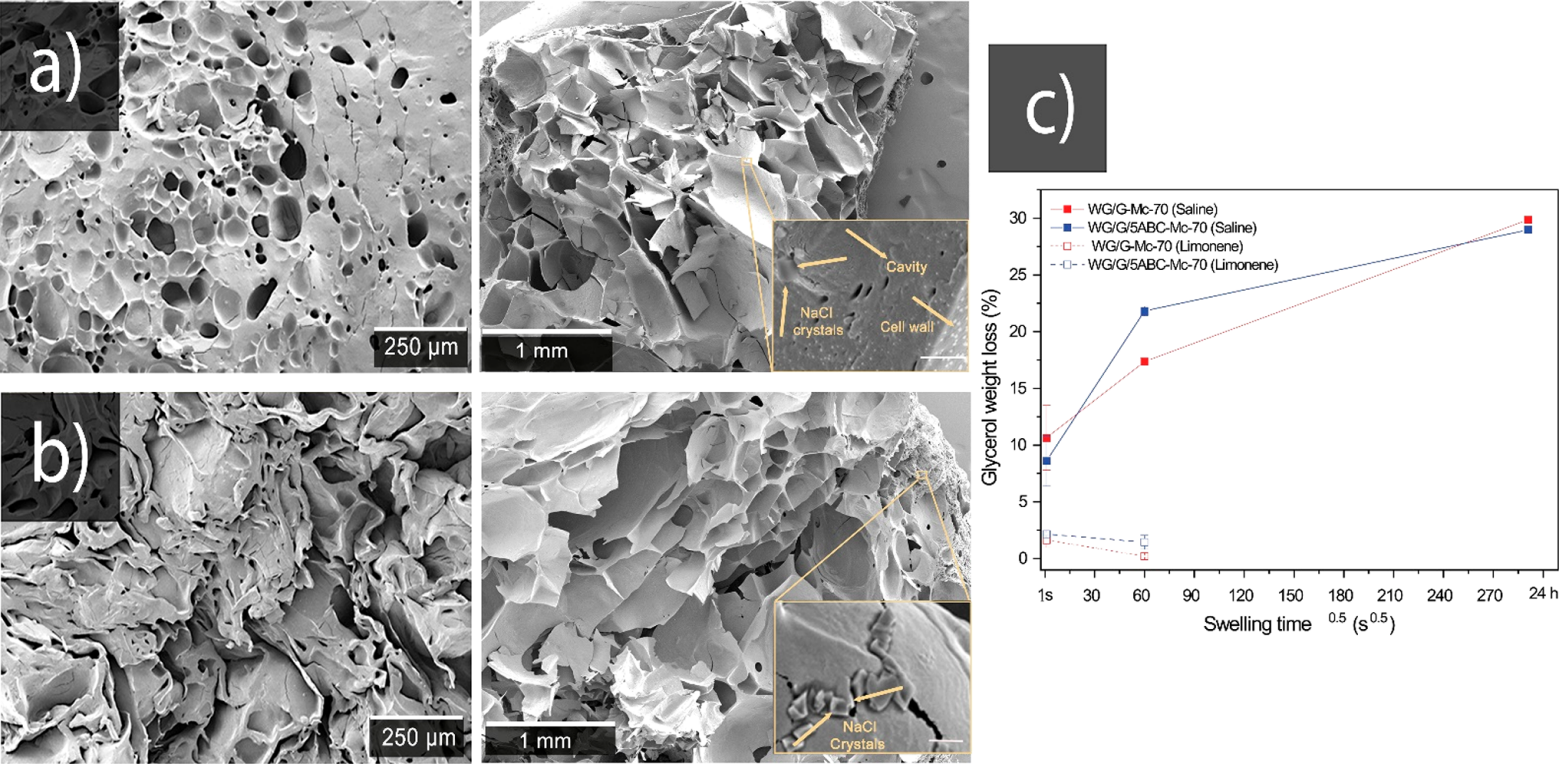
(a) WG/G-Mc-70 before (left) and after (right) 24 h swelling. (b) Corresponding images for WG/G/5ABC-Mc-70. (c) Glycerol weight loss (in absolute percentage) in WG/G-Mc-70 and WG/G/5ABC-Mc-70 samples during immersion in saline and limonene. The inset images show embedded salt crystals in the pore wall. The bars in the insets correspond to 500 nm.

It should be noted that there was a continuous and essentially complete loss of glycerol to the saline solution up to 24 h, as revealed gravimetrically (Figure 6c). The glycerol features in the IR spectrum were also absent after the 24 h period (Figure S3). Hence, if the 24 h saline uptake measurement is compensated for the parallel loss of glycerol, the sample that absorbed most saline, WG/G/5ABC-Mc (Figure 6b), would have a theoretical uptake of about 215 wt %, rather than 140% (Figure 5c). This compares favorably with the previous work on extruded WG/water mixtures, where uptake of almost 300% of pure water was observed;^39^ as mentioned above, the saline uptake is generally significantly smaller (on the order of 4–5 times) than for water.^39^ The ability of saline, due primarily to its polarity, to extract and dissolve glycerol^49,50^ was not, as expected, seen in the case of the nonpolar limonene (Figure 6c).

To visualize the saline uptake features, the extrudates produced at low temperature in the mini-compounder (WG/G-Mc-70 and WG/G/5ABC-Mc-70) were swollen in saline for 24 h freeze-dried and cryo-fractured (Figure 6a,b). The much larger pores in the swollen samples (on the order of 200 and 300 μm, respectively) indicated that the saline solution readily entered into the pores. It is also clearly shown that the “collapsed” pores in the dried WG/G/5ABC-Mc-70 sample (Figure 6b, left) opened up/expanded again during the saline uptake (Figure 6b, right). The saline diffused into the cell walls is shown in Figure 6 with inset, where the salt crystals (NaCl) can be found embedded in the cell wall. Accordingly, EDS showed that these crystals were rich in chlorine (Figure S4).

To determine the effects of the foaming agent on the saline uptake kinetics, the uptake kinetics were modeled/evaluated for the microcompounded samples without and with ABC (WG/G-Mc-70 and WG/G/5ABC-Mc-70). The saline diffusion in the WG foams was considered one-dimensional since the length of the cylindrical foam samples was more than 10× the diameter.^33,51^ Mathematically the water uptake was described by Fick’s second law of diffusion for a cylinder shape:
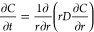
6where *C* is the solute concentration (g solute/g foam), *D*is its diffusion coefficient (cm^2^/s) , and *r* is the radial position. The concentration profiles described by eq 6 were generated by first discretizing (in cylindrical coordinates) the spatial derivatives of the partial differential equation, eq 6, and then solving the resulting ordinary differential equation (ODE) with Matlabs intrinsic ODE solver ode15s. Because of the complexity of the uptake kinetics/curves, only the initial part of the uptake was considered to estimate the diffusion kinetics. Further, the diffusion coefficient was approximated as a constant, and the sample geometry was considered unchanged during the uptake. It should also be noted, as mentioned above, that glycerol loss occurred in parallel with the saline uptake. With all these boundary conditions imposed, it was only meaningful to compare the systems and have an order of magnitude assessment of the initial more rapid uptake (i.e., within the first 30–40% uptake). As observed in Figure S5, the uptake curves, with or without the ABC foaming agent, were quite different at the later stages of the uptake. The more gradual uptake at longer periods in the ABC-foamed sample was probably due to the slow expansion of the collapsed cells (Figure 2b). The initial uptake of the ABC sample was about 40× faster (*D* = 1.60 × 10^–5^ cm^2^/s) than that of the sample without ABC (*D* = 4.33 × 10^–6^ cm^2^/s), which is in accordance with the lower density of the former sample (Table 2).

## 4 Conclusions

The production of wheat gluten high-density biobased foams (densities between 700 and 1100 kg/m^3^), without adding water, was established as an industrially upscalable extrusion processes. The foams contained both open and closed pores, and the largest pore size was obtained when adding ammonium bicarbonate (ABC) as a blowing agent. The use of ABC had benefits compared to the more commonly used sodium bicarbonate blowing agent; its low decomposition temperature made it possible to produce foams at a temperature (70 °C) well below the temperature region where protein aggregation occurs, reducing the risk of a rapidly increasing viscosity in the extrusion process. In contrast to ABC, SBC contributes to alkaline decomposition products yielding higher pH, which triggers thiol oxidation and a more extensive disulfide cross-linking and protein aggregation. SBC is effective in the presence of water, also allowing a lower processing temperature to be used. However, the approach used here was to use dry extrusion (without adding water). A porous foam was, however, also achieved without adding any foaming agent. Even though the WG powder was dried in a desiccator before mixing it with glycerol and adding it quickly to the extruder, the small WG powder particles were likely to take up moisture to the extent that the steam created pores in the extruder. However, the sample without blowing agent that stood out in terms of low density (WG/G-Se-70), had the largest content of closed pores and relatively little open pores, which led to a low saline uptake. In addition, in the most interesting processing conditions (the 70 °C case, with low protein aggregation), the largest uptake of limonene was observed in the ABC system.

To conclude, the results here showed potential in the extrusion foaming technique for protein-based materials, depending on the processing parameters and agents added, and that extruded protein foams can compete with less sustainable petroleum-based polymer foams. They provide possibly a solution where microplastic problems can be minimized and where the raw material comes as coproducts/byproducts from existing industrial processes. Wheat gluten, in particular, is considered a byproduct in some parts of the world, and the glycerol plasticizer is a large byproduct from biodiesel production.

## Supporting Information Available

FE-SEM image of the wheat gluten powder and particle size distribution (Figure S1); Full IR spectra and the amide I region of samples produced with the single-screw extruder (Figure S2); Full IR spectra pointing out the glycerol-related peaks after 24 h swelling (Figure S3); SEM image showing embedded NaCl crystals and elemental analysis by EDS (Figure S4); Experimental and simulated mass uptake versus square root of time of the foams (Figure S5); Content of different molecular features from IR spectra in the amide I region (Table S1) (PDF)
