# Supplementary material for: Sustainable Wheat Protein Biofoams: Dry Upscalable Extrusion at Low Temperature
Source: Biomacromolecules. 2022 Nov 9;23(12):5116–26. doi: 10.1021/acs.biomac.2c00953 (PMC9748940; doi:10.1021/acs.biomac.2c00953)
Supplement: Supplementary file 1 — bm2c00953_si_001.pdf [file bm2c00953_si_001.pdf]

Supplementary information

Sustainable wheat protein biofoams – dry upscalable extrusion at low temperature

Mercedes A. Bettelli,[1] Antonio J. Capezza,[1] Fritjof Nilsson,[2] Eva Johansson,[3]

Richard T. Olsson,[1] Mikael S. Hedenqvist,[1*]

1 Department of Fibre and Polymer Technology, Polymeric Materials Division, School of Engineering Sciences in Chemistry, Biotechnology and Health. KTH Royal Institute of Technology, Stockholm 10044, Sweden.

2 FSCN research centre, Mid Sweden University, Sundsvall 85170, Sweden.

3 Department of Plant Breeding, SLU Swedish University of Agriculture Sciences, Alnarp, SE-23053, Sweden

* Correspondence: mikaelhe@kth.se; Tel: +46-706507645

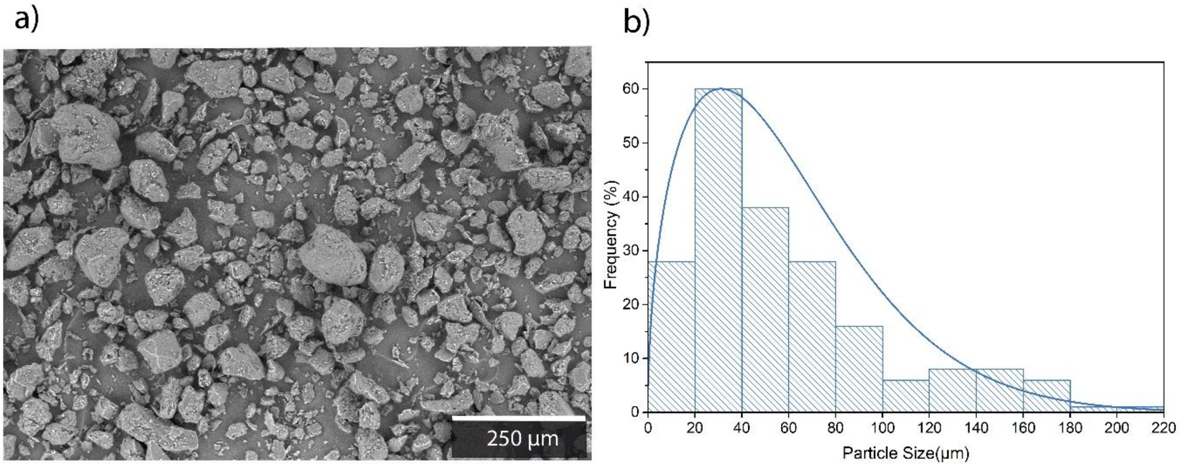

Figure S1: a) FE-SEM image of the wheat gluten powder, b) particle size distribution.

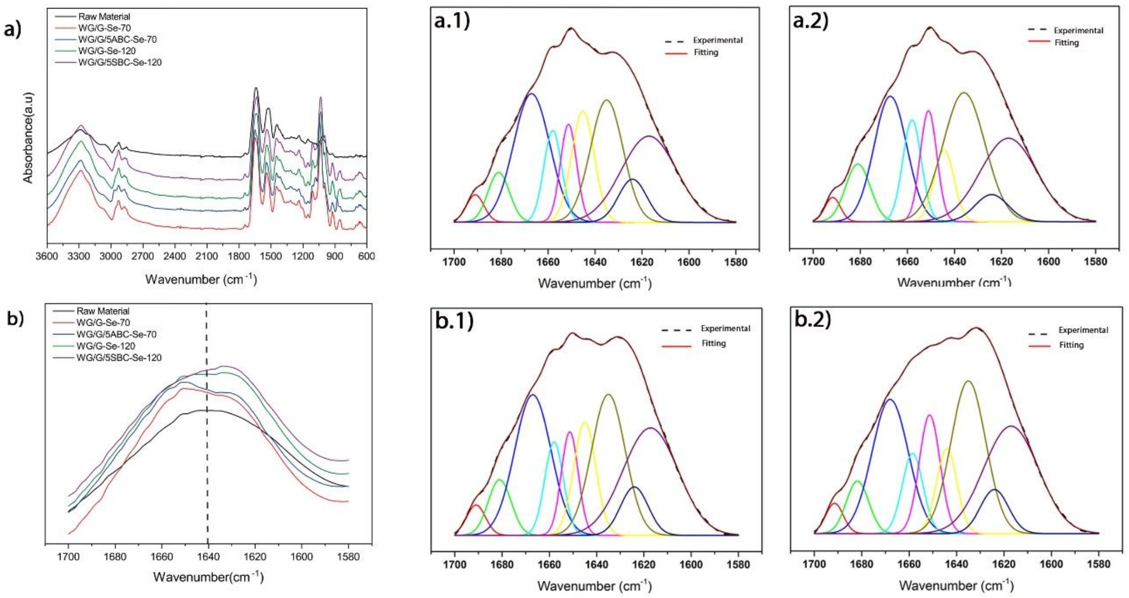

Figure S2. a) Full IR spectra and b) the amide I region of samples produced with the single-screw extruder. The deconvoluted amide I region with the corresponding fitted curves for: (a.1) WG/G-Se-70, (a.2) WG/G/5ABC-Se-70, (b.1) WG/G-Se-120 and (b.2) WG/G/5SBC-Se-120.

Table S1: Content of different molecular features from IR Spectra in the Amide I region

Method II: Extruder					
**λ (cm^{-1})**	**Assignment**	**WG/G-Se-70 (%)**	**WG/G/5ABC-Se-70 (%)**	**WG/G-Se-120 (%)**	**WG/G/5SBC-Se-120 (%)**
1618, 1625	β-sheets strongly bonded	27.2	25.3	29.0	28.6
1634, 1681	β-sheets weakly bonded	23.1	31.0	24.2	26.1
1644, 1651, 1658	α-helixes and random coil	26.3	23.5	24.0	23.5
1667, 1692	β-turns	23.4	20.3	22.8	21.8

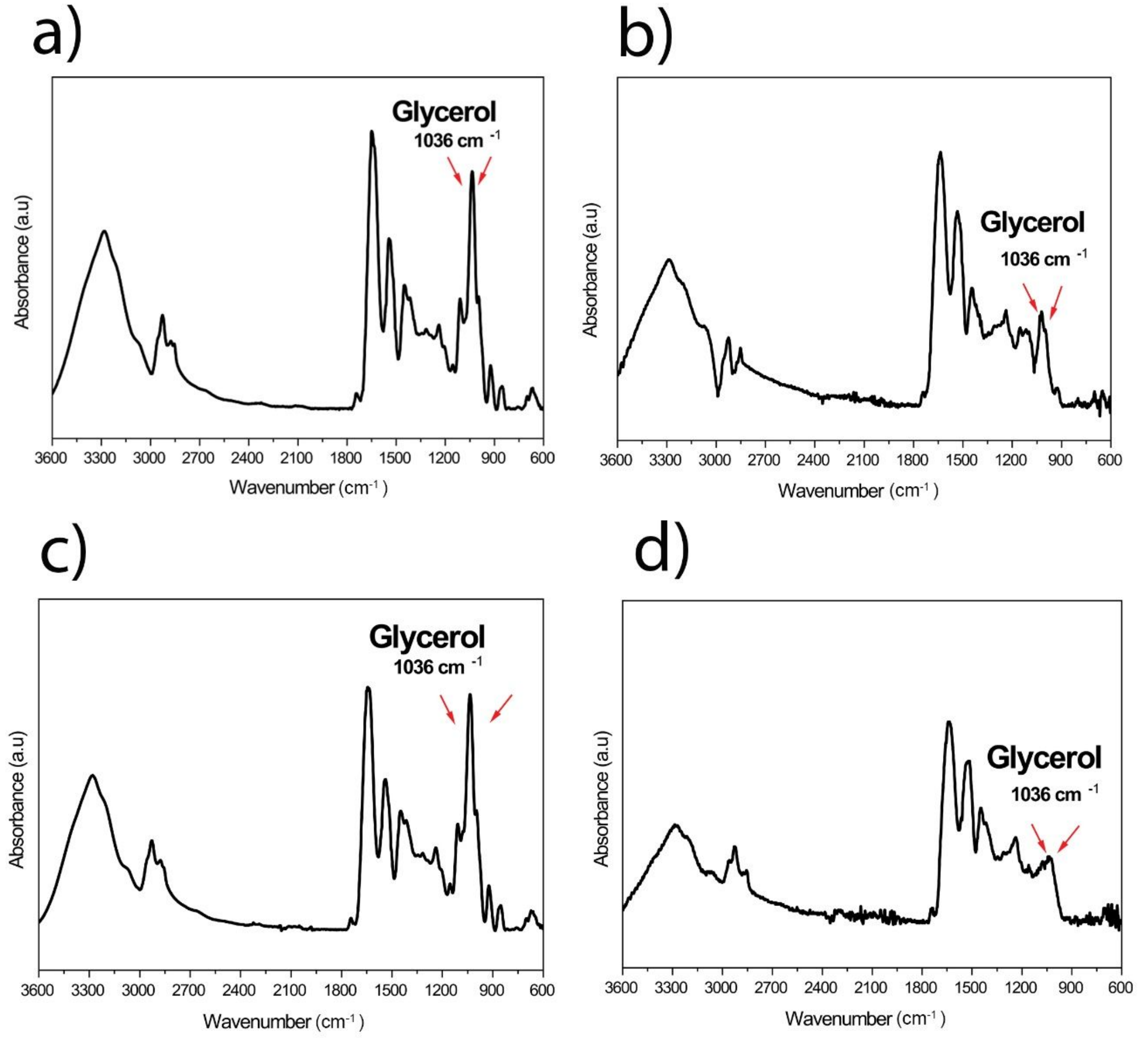

Figure S3: a) Full IR spectra pointing out the glycerol-related peaks: a) WG/G-Mc-70, b) WG/G-Mc-70 (after 24 h swelling), c) WG/G/5ABC-Mc-70, (d) WG/G/5ABC-Mc-70 (after 24 h swelling).

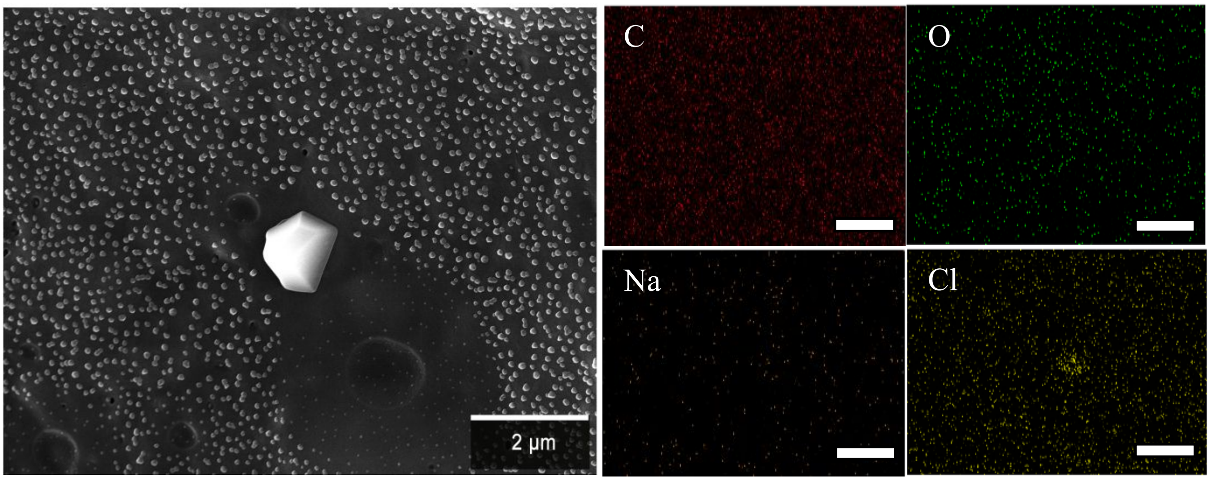

Figure S4: SEM image showing a relatively large NaCl crystal (in the middle) in the WG/G-Mc-70 foam (left). EDS images showing the presence of the elements C, O, Na and Cl. The scale bars are 2.5 µm long (right).

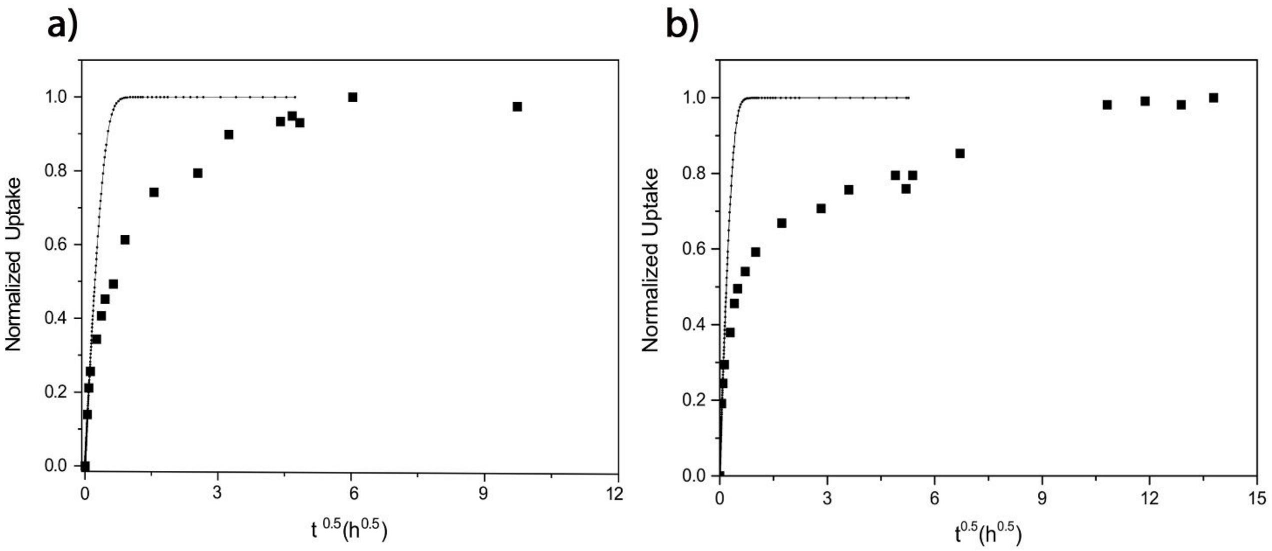

Figure S5: Experimental and simulated mass uptake versus square root of time of the foams: a) WG/G-Mc-70 and b) WG/G/5ABC-Mc-70.
